# Method for lysis and paper-based elution-free DNA extraction with colourimetric isothermal amplification

**DOI:** 10.1038/s41598-024-59763-4

**Published:** 2024-06-24

**Authors:** Soo Min Lee, Egan H. Doeven, Dan Yuan, Rosanne M. Guijt

**Affiliations:** 1 Centre for Regional and Rural Futures (CeRRF), Deakin University, Locked Bag 20000, Geelong, VIC 3220 Australia; 2 School of Life and Environmental Sciences, Faculty of Science, Engineering and Built Environment, Deakin University, Waurn Ponds, VIC 3216 Australia; 3 School of Mechanical and Mining Engineering, The University of Queensland, Brisbane, QLD 4072 Australia

**Keywords:** Paper-based DNA extraction, PASAP, Nucleic acid amplification test (NAAT), Elution-free sample preparation, Colourimetric loop-mediated isothermal amplification (cLAMP), *E. coli* detection, Milk, Point-of-need testing, PONT, Analytical biochemistry, Lab-on-a-chip, Sensors and probes, Bioanalytical chemistry, Lab-on-a-chip, Microfluidics, Sensors

## Abstract

Nucleic acid amplification testing has great potential for point-of-need diagnostic testing with high detection sensitivity and specificity. Current sample preparation is limited by a tedious workflow requiring multiple steps, reagents and instrumentation, hampering nucleic acid testing at point of need. In this study, we present the use of mixed cellulose ester (MCE) paper for DNA binding by ionic interaction under molecular crowding conditions and fluid transport by wicking. The poly(ethylene) glycol-based (PEG) reagent simultaneously provides the high pH for alkaline lysis and crowding effects for ionic binding of the DNA under high salt conditions. In this study, we introduce Paper-based Abridged Solid-Phase Extraction with Alkaline Poly(ethylene) Glycol Lysis (PASAP). The anionic mixed cellulose ester (MCE) paper is used as solid phase and allows for fluid transport by wicking, eliminating the need for pipetting skills and the use of a magnet to retain beads. Following the release of DNA from the cells due to the lytic activity of the PASAP solution, the DNA binds to the anionic surface of the MCE paper, concentrating at the bottom while the sample matrix is transported towards the top by wicking. The paper was washed by dipping it in 40% isopropanol for 10 s. After air-drying for 30 s, the bottom section of the paper (3 mm × 4 mm) was snapped off using the cap of a PCR tube and immersed in the colourimetric loop-mediated isothermal amplification (cLAMP) solution for direct amplification and colourimetric detection. The total sample processing was completed in 15 min and ready for amplification. cLAMP enabled the detection of 10^2^ CFU/mL of *Escherichia coli* (*E. coli*) from culture media and the detection of *E. coli* in milk < 10^3^ CFU/mL (10 CFU) after incubation at 68 °C for 60 min, demonstrating applicability of the method to complex biological samples.

## Introduction

Nucleic acid amplification tests (NAATs) have gained popularity due to their high sensitivity and specificity for identifying target species in samples of interest. Research has focused on integrating NAAT into portable devices to meet the increasing demand for point-of-need testing (PONT)^1^. In parallel, paper analytical devices (PADs) have been demonstrated to provide an affordable substrate for chemical and biological assays due to accessible manufacture, low cost, and ease of disposal^2–5^. PADs can be as simple as the popular lateral flow-based immunoassays (LFA) that patients can self-administer for diagnostic tests, such as for pregnancy and coronavirus (COVID-19). In addition to clinical settings, PONT is also important in warranting food safety^6^, with recent progress in LFA and PAD-based technologies applied to chemical and biological food safety reviewed elsewhere^7,8^. The challenge in developing 'sample-to-answer' PADs for NAATs is in sample preparation^9^ because NAATs requires more extensive processing compared to immunoassays typically used in LFA tests to prevent attenuation of the amplification reaction. In the traditional, laboratory-based workflow, sample preparation for NAAT comprises two main steps: lysis and solid-phase extraction (binding, washing, and elution).

In the simplest form, PADs have been used for the detection of amplification products generated off-device, eliminating the need for detection instrumentation. More recently, more advanced PADs have used paper as a carrier for dried reagents that—following rehydration—can be used for isothermal amplification by loop-mediated isothermal amplification (LAMP) and recombinase polymerase amplification (RPA) on the paper membrane^4,10,11^. Inspired by the traditional art of origami, folded PADs were introduced to bring complementary functionalised areas in close proximity to facilitate a sequence of sample preparation steps on a compact device^11–17^. Various papers have been utilised for PADs, including glass fibre^18^, polyethersulfone (PES) filters^11,19^, and Flinders Technology Associates (FTA) cards^3^. In recently reported advances, a 3D-printed rotational device was developed to minimise manual handling by accommodating an assay using a glass fibre pad for Nucleic Acid (NA) capture and RT-LAMP using freeze-dried reagents^18^. Another work reported a multi-layer, multi-material PAD where the NAs were captured on a PES filter, followed by LAMP on a 3 mm × 3 mm glass fibre pad after reconstituted dried reagents, including hydroxynaphthol blue for colourimetric detection^19^. In other work, FTA cards were placed in a polymer fluidic cartridge, allowing for the transfer of NAs on the paper disk to a vial for amplification to detect SARS-Cov-2-virus and *Helicobacter pylori* down to 4 × 10^2^ copies/mL by fluorescence detection^3^.

In PADs, however, lysis is often conducted off-device, and a lengthy drying step may be required before eluting the captured DNA. Additionally, complex PADs that embody multiple foldings and materials to accommodate the processing steps, impose a need for highly developed skills during manufacture and operation and associated increased cost and risk of contamination with manual handling during manufacture and use. Commercially available mixed cellulose ester (MCE) membrane is a paper consisting of about 70% cellulose nitrate and 30% cellulose acetate and is negatively charged at neutral pH. The paper is employed, for example to filter lactic acid bacteria to detect beer spoilage using Barocylcer™ treatment^20^ or to capture environmental DNA from water samples where MCE outperforms PES in capturing and preservation capacity^21^, probably due to the ability of MCE paper to resist DNA digestion by DNase I and proteinase K^22^.

**Figure 1:**
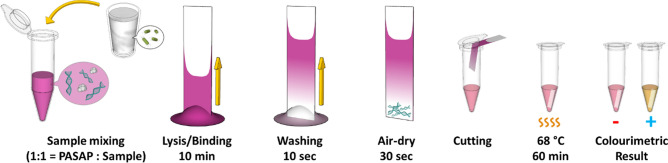
Schematic illustration of the PASAP method for simultaneous cell lysis and DNA binding on paper. The sample and the PASAP solution are mixed in a 1:1 ratio, and 20 µL of the mixture is absorbed by the MCE paper, wicking it from the short end. Under crowding conditions, the DNA binds to the bottom section of the paper. After 10 min the 20 μL is absorbed and the paper tip is washed with 40% IPA for 10 s and allowed to air-dry for 30 s. The tip of the paper is snapped off using a cap of a PCR tube and used for direct on-paper cLAMP for 60 min. The amplification results are visualised by a colour change of the LAMP solution (pink = negative, yellow = positive).

Here, we propose to use the DNA capturing capacity of MCE paper as stationary phase during sample preparation. We recently reported Abridged Solid-Phase Extraction with Alkaline Poly(ethylene) Glycol Lysis (ASAP), introducing a single reagent that combines cell lysis and DNA immobilisation at the anionic surface of paramagnetic particles before on-bead amplification^23^. The method used the concentration-dependent alkalinity of poly(ethylene) glycol (PEG) to provide the alkalinity required for alkaline lysis and solubilisation of the NAs as well as its rapid decrease in pH upon dilution to bring the sample solution pH into the realms of the buffer capacity of amplification buffers^24^. Simultaneously, the molecular crowding effect of PEG facilitated DNA precipitation onto the anionic support under high salt conditions. However, paramagnetic beads used in ASAP led to some experimental challenges, as bead recovery was compromised by the relatively high viscosity of the PEG—also limiting the sample volume—and the binding capacity limited by the bead mass tolerated during amplification. Here, the method is advanced by substituting the beads with paper for paper-based ASAP (PASAP), as schematically illustrated in Fig. 1. The reagent was re-optimised to bind the DNA to a mixed MCE paper under high salt conditions, which after a washing step was directly deposited in a PCR tube for amplification. Polymerase chain reaction (PCR)—the gold stand for molecular diagnostis-and LAMP were both evaluated. PCR has limited compatibility with use in low resource settings due to the need for thermal cycling, whereas LAMP is isothermal and can be combined with visual detection incorporating a colorimetric reagent to confirm amplification of the target^25^. Following experimentally determined incompatibility of PCR with amplification on the MCE paper, colorimetric LAMP (cLAMP) was selected for amplification in presence of the MCE paper. The PASAP method was applied to do DNA sample preparation for the detection of *Escherichia coli* (*E. coli*) in milk, a complex biological matrix comprising organic and inorganic small molecules, proteins, and lipids aggregated in a colloidal suspension^26^. The optimised method is conducted as a four-step assay: (1) wicking of the lysate into the paper, (2) a 10-s wash using 40% isopropanol (IPA), (3) 30-s air-drying, and (4) cLAMP following the release of the tip of the paper into the PCR tube by snapping the brittle paper using the cap of the tube. Detection of the target DNA was achieved through the colour change from pink (pH > 8.2) to yellow (pH  < 6.8) due to decreased pH as H^+^ ions are generated during amplification^2^.

The PASAP method resolves major challenges in sample preparation for NAAT by reducing the number of reagents and processing steps. It does not rely on external equipment other than a pipette (and a heater for amplification). After 15 min, a PCR tube containing the paper is ready to be loaded into a suitable heating system. The method produces minimal plastic waste (two PCR tubes and two pipette tips), eliminating the need for a magnet and associated handling challenges in comparison with the ASAP method. Isothermal amplification can be done in a thermocycler, or using a battery-powered heater. The PASAP method allowed for the detection of *E. coli* in milk samples with a limit of detection (LOD) of 10^3^ colony forming unit (CFU)/mL (40% success rate at 10^2^ CFU/mL), which is below the maximum allowable plate count of 10^4^–10^5^ CFU/mL for raw and pasteurised milk intended for further processing^27^. Combined with a battery-powered heater, the approach has potential for rapid and affordable NA-PONT for applications including food safety.

## Methods

### Materials

Absolute ethanol, IPA, potassium hydroxide (KOH), and sodium chloride (NaCl) were obtained from Chem Supply Australia (Gillman, Australia). Luria–Bertani Broth (LB), agar, and PEG 8000 were purchased from PhytoTechnology Laboratories (Lenexam, USA). Ethylenediaminetetraacetic acid (EDTA) solution (03690), nuclease-free water (W4502), phosphate-buffered saline (PBS), Tween 20 (P7949), 2-dodecanol (D221503), and 1-decanol (239763) were obtained from Sigma-Aldrich® (Macquarie Park, Australia). Phenol red (32661) was obtained from Fluka (ChemSupply Australia, Gilwell, Australia). Hydrophilic MCE membrane filters with 1.2 µm (RAWP04700), 5.0 µm (SMWP04700), and 8.0 µm pore size (SCWP04700) were purchased from MF-Millipore™ Membrane Filter (Millipore, North Ryde, Australia) and cut into 45 mm long strips. Milli-Q water was obtained from a Cascada™ Lab water purification system (Pall Australia, Melbourne, Australia) and autoclaved using Pratika S20 from Siltex (Bentley East, Australia) before its use. Luna® Universal qPCR Master mix (M3003) and WarmStart® Colorimetric LAMP 2 × Master Mix (DNA & RNA) (M1800) were purchased from New England Biolabs (Nottinghill, Australia). Molecular grade agarose (BIO-41025) was obtained from Bioline (Eveleigh, Australia).

Purified *E. coli* genomic DNA (gDNA) was obtained using an ISOLATE II Genomic DNA extraction kit (Bioline, Eveleigh, Australia) following the manufacturer's instructions, and the eluate was stored in a 1.5-mL DNA LoBind® Tube (Eppendorf, part # 0030108051, Macquarie Park, Australia) at – 20 °C until further use.

### PASAP

The 2 × PASAP solution was prepared by mixing 0.1753 g of NaCl, 0.3 g of 50% (w/v) PEG 8000, 2 µL of 100% Tween 20, 1 µL of 0.5 M EDTA, 100 µL of 20 mM phenol red, and water up to 1 mL. To perform PASAP, 49 µL of the PASAP stock solution, 1 µL of 700 mM KOH, and 50 µL sample were briefly mixed in a 1.5-mL centrifuge tube. A 20 µL of this lysate was pipetted on a petri dish surface and absorbed by a MCE paper strip (4 mm × 45 mm), a process that takes about 10 min, after which the tip of the paper was placed in a 20 µL drop of 40% IPA for 10 s for washing. The paper was then air-dried for ~ 30 s at room temperature. The tip of the paper was laid flat on a PCR tube and snapped off by closing the lid, dropping the ~ 4 mm × 3 mm section in 20 µL LAMP reaction solution. The brittle MCE paper easily snaps, making the pressure applied by the lid of a PCR tube sufficient to cut off the tip. Finally, cLAMP is conducted at 68 °C for 60 min without removal of the paper.

### Real-time PCR (qPCR) analysis

Amplification and real-time fluorescence readings were conducted on the CFX Connect Real-Time PCR System (Bio-Rad). The qPCR reactions included 10 µL of Luna® Universal qPCR Master mix, 0.25 µL of 10 µM each forward and reverse primer, 5 µL of DNA template, and 4.5 µL of nuclease-free water, resulting in a 20 µL final reaction volume. Forward and reverse primers previously designed to target the Escherichia phage Lambda gene in *E. coli* strain BL21 were used^23^. Thermal cycling conditions were 1 min at 95 °C for initial denaturation, followed by 40 cycles at 95 °C for 15 s and 60 °C for 30 s, with a melt-curve analysis from 65 to 95 °C at 0.5 °C intervals.

The Cq values obtained from qPCR amplification can be used to calculate the DNA concentration using a previously constructed standard curve^23^. However, as most amplifiactions were conducted under attenuating conditions, all qPCR data is reported as number of cycles (Cq).

### cLAMP reactions

All LAMP primers used in this study were taken based on an earlier literature report^28^ and purchased from Integrated DNA Technologies (IDT). Before the LAMP reaction, a 10 × primer mix containing 1.6 µM of each forward inner primer (FIP) and backward inner primer (BIP), 0.2 µM of each forward outer primer (F3) and backward outer primer (B3), and 0.4 µM of each forward loop primer (LF) and backward loop primer (BF) was prepared. The cLAMP reaction contained 10 µL of WarmStart® Colorimetric LAMP 2 × Master Mix (DNA & RNA), 2 µL of 10 × primer mix, and 2 µL of nuclease-free water, making the final volume of 20 µL per reaction. Once the DNA-carrying MCE paper was immersed in the LAMP mixture, the PCR tubes were incubated at 68 °C for 60 min in Eppendorf mastercycler × 50 s, in agreement with earlier reports^25–28^. The presence of amplicons was confirmed by gel electrophoresis on a 3% (w/v) agarose gel.

The potential for in-field use was confirmed using a 12 V heater consisting of an Arduino Nano microcontroller (Seeed Studio), running a PID algorithm which controlled a 12 V 2A cartridge heater via switching a transistor. Temperature feedback was provided by a DS18B20 digital temperature probe. The temperature probe and heater cartridge were mounted in drilled holes within an aluminium block also containing recesses for 8 PCR tubes. Using a laptop, an interface designed in MegunoLink was used to maintain the temperature at 68 °C, start and stop the heating process, and view the temperature of the block over time graphically. The heater system was powered by a 12 V lab power supply, or a portable power bank with a USB-C power-delivery (Comsol 25,600 mAh 100 W power bank, Officeworks, Australia). Arduino and interface code are available on request. As the portable system lacked a cooled lid, a drop of silicone oil was added on top of the LAMP mixture to prevent evaporation. Images of experimental set-up and preliminary results are available in Fig. S9.

### *E. coli* detection from milk samples

A colony of *E. coli* BL21 was cultured in 10 mL of sterile LB broth placed on an orbital shaker (Thermoline Scientific Equipment Pty Ltd, Model: TU-400, Wetherill Park, Australia) overnight at 37 °C and 150 rpm. Subsequently, 100 µL of the liquid culture was transferred to fresh LB, incubated at 37 °C and 220 rpm until the OD_600_ (optical density at 600 nm) reached 0.5 measured by a GENESYS™ 30 Visible Spectrophotometer (Thermo Fisher Scientific, Scoresby, Australia). Next, 1 mL of the cell suspension was dispensed into a sterilised 1.5 mL microcentrifuge tube (NEST®, cat. No. 615001, Adelab Scientific, Thebarton, Australia) and centrifuged for 2 min at 8000 rpm using a PicoTM 21 centrifuge (Thermo Fisher Scientific, Scoresby, Australia). The resulting cell pellet underwent three washes with a sterile PBS solution. Finally, the washed cells were resuspended in 1 mL of PBS and promptly stored at – 20 °C for future use.

Skim long-life milk (Coles Heat Treated Australian Milk, Coles Group limited, Hawthorn East, Australia), containing 120 mg calcium, 40 mg sodium, 4.8 g carbohydrate, 3.3 g protein, and less than 1 g of saturated fat, was purchased from a local supermarket. To perform *E. coli* detection in milk, 1 mL of the cultured *E. coli* was centrifuged to obtain a cell pellet and resuspended in 1 mL of tenfold diluted skim milk. This spiked milk was then serially diluted with ten-fold diluted milk with the final concentrations ranging from 10^6^ to 10^1^ CFU/mL and used for PASAP.

### Statistical and image analysis

The data analysis for qPCR was conducted using Bio-Rad CFX Manager 3.1 software, with Cq determination in single threshold mode. Statistical analysis and graphing were executed using OriginPro 2022b Learning Edition. Each data point represents a minimum sample size of 3 (n = 3), and paired comparison plots were generated with Tukey test and significance differences defined as *p* ≤ 0.05 (*) and *p* ≤ 0.01 (**) through the post-hoc method using Two-way ANOVA.

To analyse photographs following cLAMP, raw images were subjected to RGB splitting, and the green and blue channels' grey values were measured using ImageJ following a published study^29^. Then, the mean intensity values of blue were subtracted from green, and the difference was plotted.

## Results and discussion

### On-paper amplification

The compatibility of commonly used amplification approaches—PCR and LAMP—with MCE paper was investigated, using MCE paper because of its negative charge, providing potential DNA binding capacity under crowding conditions and quick drying owing to the thin film (130 µm). In our earlier work, the viscosity of the ASAP solution provided a challenge in magnetically recovering the beads during DNA extraction^23^. With the ambition of using the wicking by MCE paper for fluid transport, the wicking time of the viscous solution was examined with MCE paper with 1.2, 5.0, and 8.0 µm pore size, with the results presented in Fig. S1. As expected for the limited height to be wicked^30^, smaller pores correlated with higher resistance and hence lower flow rates, with the 1.2 µm MCE paper showing the slowest speed, while the rates for 5.0 and 8.0 µm were comparable. Thus, 8.0 µm MCE paper was selected for faster processing.

After optimisation on pore size, 8.0 µm pore size, MCE paper (3 mm × 4 mm) was introduced into PCR vials containing target DNA, primers, and an amplification mix, aiming for fluorescence detection of the amplification product using SYBR green as an intercalating dye. No fluorescence could be detected during qPCR and qLAMP (Figs. S2 and S3). However, amplicons were detected by gel electrophoresis following LAMP but not PCR, indicating the paper inhibited both amplification and detection during qPCR but only interfered with the fluorescence detection in qLAMP. Combined with an expected loss in sensitivity at higher salt levels because of an increase in dissociation constant (K_d_) of SYBR green for DNA^1,29^, fluorescence detection was deemed unsuitable for the targeted extraction following PASAP. cLAMP has become a popular choice for in-field testing for non-quantitative detection^31^. Because the advantages of isothermal amplification include elimination of thermocycling and better salt tolerance of *Bst* 2.0 polymerase (used in LAMP amplification for strand displacement activity) than *Taq* polymerase used in PCR (complete inhibition at 100 mM vs 40 mM NaCl)^32^, making LAMP the preferred amplification approach for PASAP.

ASAP method combined lysis and extraction with direct, on-bead DNA amplification. The approach combines the reversible binding of NAs in the presence of PEG for solid phase extraction onto paramagnetic beads, an approach conceptually similar to solid phase reversible immobilisation (SPRI)^23^, with DNA binding facilitated by the crowding effects of PEG under high salt conditions. Here, the aim is to use MCE paper to act as a stationary phase and fluid handling by wicking.

To confirm binding of the DNA to the MCE paper, a strip of MCE paper was used to wick 20 µL of a sample containing 10 µL purified gDNA and 10 µL 15% PEG 8000 and 3.5 mM KOH in the presence and absence of 1 M NaCl. After wicking the 20 µL sample following the insertion of the short edge into the droplet, the strip was washed with 70% ethanol before being cut into about 3 mm × 4 mm long at the top (TOP), middle (MID), and bottom (BTM) sections of the paper. Each of these was placed in 20 µL of water to elute the gDNA, after which 5 µL of the eluate was used for qPCR.

**Figure 2:**
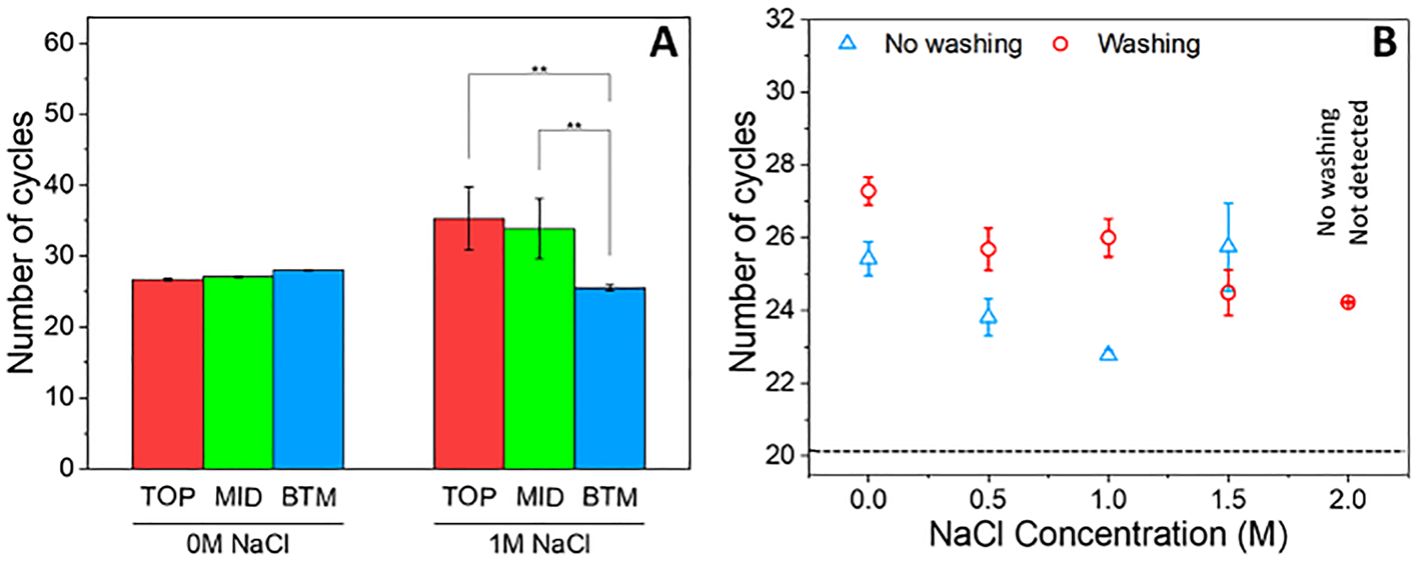
Extraction of gDNA onto MCE paper. (**A**) qPCR cycle number for gDNA in the eluate for the TOP (red), MID (green) and BTM (blue) sections of a 4 mm wide, 45 mm long strip of MCE paper. Data reported in absence of NaCl (0 M) and in presence of 1 M NaCl. BTM refers to the bottom 15 mm, MID to the middle section of 15 mm and TOP to the remaining 15 mm of the strip. (**B**) qPCR cycle number for gDNA in the eluate from the BTM for NaCl concentration increasing from 0 to 2 M in the PASAP solution. Eluates were analysed with (red circle) and without (blue triangle) a washing step with 70% ethanol. Black dashed line shows the Cq value for the purified gDNA input.

When no salt was present, the larger gDNA concentrations found in the eluates from the MID and TOP sections indicating that the DNA was carried up the MCE paper by the wicking of the aqueous solution by the hydrophilic porous material (Fig. 2A). In absence of the high ionic strength required for DNA binding using 0 M NaCl, no significant difference in the Cq values between the TOP, MID, and BTM section of the paper were obtained. As expected, when the ionic strength was increased using 1 M NaCl, the DNA was retained in the bottom section of the MCE paper. The high Na^+^ concentration facilitates electrostatic bonding between the slightly anionic surface and DNA^33,34^, partially neutralising the surface charge and attracting the anionic DNA, noting charge density is reduced under the crowding conditions in the presence of PEG 8000, inducing DNA precipitation at the surface. The concentration of the gDNA in the tip of the paper strip in the presence of 1 M NaCl demonstrates the potential of using MCE paper for DNA extraction under ASAP conditions.

Encouraged by these results, the effect of NaCl concentration was investigated using a reagent combining 15% PEG 8000 and 3.5 mM KOH with increasing NaCl concentrations (0, 0.5, 1.0, 1.5, and 2.0 M) to understand the correlation of the binding capability of paper with NaCl concentration. The gDNA in the eluate was analysed by qPCR. Under low NaCl concentrations (0—1 M) without washing, a decrease in Cq value with increasing salt concentration demonstrated the efficacy of DNA binding with increasing salt concentration (Fig. 2B). The Cq value, however, increased to 25.75 ± 1.21 at 1.5 M, and amplification was not detected at 2.0 M as a result of inhibition of amplification. The NaCl concentration in the eluate was not measured, but NaCl is known to have an inhibitory effect on *Taq* polymerase preventing amplification when [Na^+^] ≥ 40 mM^32^.

The efficacy of the washing step was investigated using 70% ethanol, commonly used in SPRI and DNA extraction protocols. Washing was performed by dipping the bottom section of the paper in a 20 µL drop of 70% ethanol for 30 s, followed by air-drying of the strip before eluting the DNA in 20 µL Milli-Q water. Disappointingly, the wash significantly increased Cq values of the eluates to 27.28, 25.68, and 26.00 for 0, 0.5, and 1.0 M NaCl, respectively, indicating considerable DNA loss during the wash (Fig. 2B). In contrast, improved Cq values of 24.49 ± 0.62 and 24.23 ± 0.03 were obtained after the wash for 1.5 and 2.0 M NaCl, correspondingly showing that the inhibitory effect of high NaCl can be alleviated by washing provided sufficient NaCl was present to ensure strong binding of the DNA to the paper.

The lowest Cq values were obtained using 1.0 M NaCl without washing. However, it is unlikely that the washing step can be eliminated when targeting complex biological samples like milk. As no improvement in sensitivity was found using 2 M NaCl compared with 1.5 M NaCl with washing for binding (Cq values 24.23 ± 0.03 vs 24.49 ± 0.62), 1.5 M NaCl with washing was selected for further method optimisation to minimise salt-induced inhibition. The increase in Cq value in comparison with input DNA (dashed line) suggests there is room for improvement, increasing binding efficiency or decreasing DNA loss and/or attenuation of amplification.

### Optimisation of the wash

The results presented above suggest that the eluting strength of 70% ethanol may be too high for it to be a suitable washing reagent. Washing reagents during DNA extraction on anionic stationary phases have included water-miscible solvents like isopropanol to remove the salt^1^. Non-polar solvents were used in the two-phase wash to limit the carryover of aqueous inhibitors but were typically added between the wash step and the final elution^28^.

**Figure 3:**
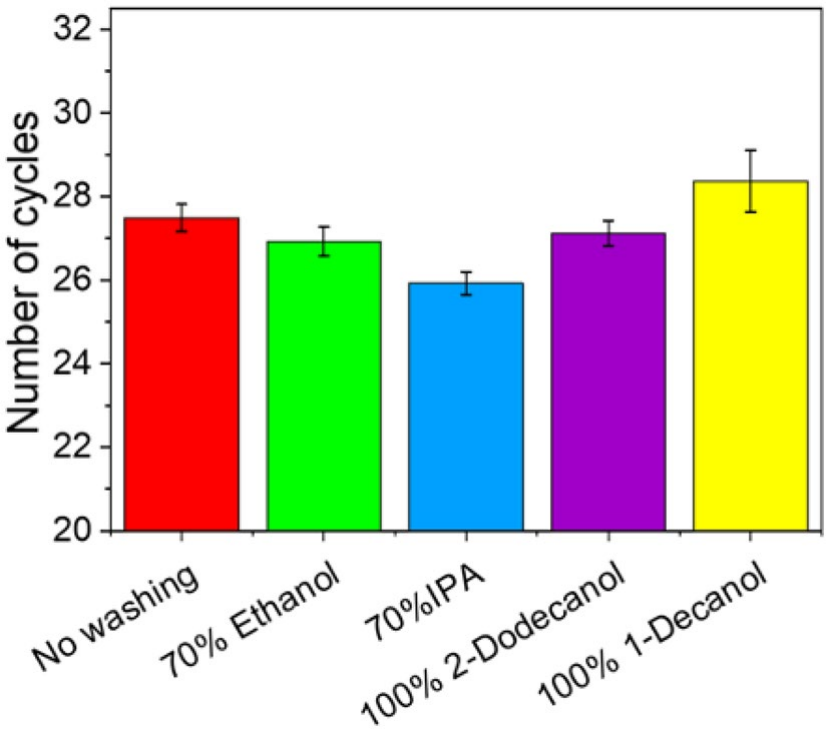
Optimisation of the washing solution. Number of qPCR cycles of the PASAP method including no washing (red), washing with 70% ethanol (green), 70% IPA (blue), 100% 2-dodecanol (purple), and 100% 1-decanol (yellow).

Four washing liquids were examined, 70% ethanol, 70% IPA and two long-chain alcohols (2-dodecanol and 1-decanol) as used in the two-phase wash^28^. In line with the experiments discussed above, 20 µL of a solution containing 10 μL each of the gDNA standard and PASAP reagent was wicked up a 45 mm long MCE strip, after which the bottom 3—4 mm was immersed in a 20 µL drop of the washing liquid for 30 s. The MCE paper was air-dried before elution into 20 µL of water. The number of qPCR cycles determined the efficacy in reducing loss and/or attenuation. As shown in Fig. 3, DNA was detected following a wash with 70% IPA, with Cq values of 25.92 ± 0.27 in the eluate. No washing increased the Cq value (27.49 ± 0.33), indicating an inhibition equivalent to a 3.3-fold DNA loss. Slightly delayed Cq values of 26.93 ± 0.33 using 70% ethanol and 27.12 ± 0.30 using 100% 2-dodecanol were found, whereas for 100% 1-decanol the Cq values were significantly increased to 28.37 ± 0.74, a higher value than *no wash*. The poor washing efficiency of both hydrophobic solvents may the due to the decreased efficacy of the salt removal owing to the poor solubility of NaCl in the washing solvent, which is vital at the high NaCl concentration used for binding.

The solubility of DNA in IPA is lower than in 70% ethanol^35^, preventing the elution of immobilised DNA while still providing good solubility for NaCl to be an effective washing agent. To optimise the capacity of the washing liquid to remove salt without eluting the DNA, the IPA content was optimised (Fig. S4), indicating that 40% IPA was most effective in mitigating attenuation with minimal processing time (10 s). For longer washes (60 s), decreasing Cq values suggest the washing efficacy of IPA > 40% increased; however, with the objective of fast sample preparation, 40% IPA was selected.

### Lysis and inhibitory effects of Tween 20 in PASAP

During ASAP, Tween 20 was required to prevent magnetic particles from aggregation but may also have contributed to the lytic activity. As the proposed PASAP method replaces the beads with the MCE paper, aggregation of particles is no longer a risk. Hence, the necessity of Tween 20 was investigated. Using *E. coli* as a biological sample, 20 µL of 10^6^ CFU/mL bacteria was resuspended in the PASAP solutions with or without 0.2% Tween 20, wicked by the MCE paper and washed with 40% IPA for 10 s or directly eluted in water for further determination of the selected washing steps for *E. coli* samples.

**Figure 4:**
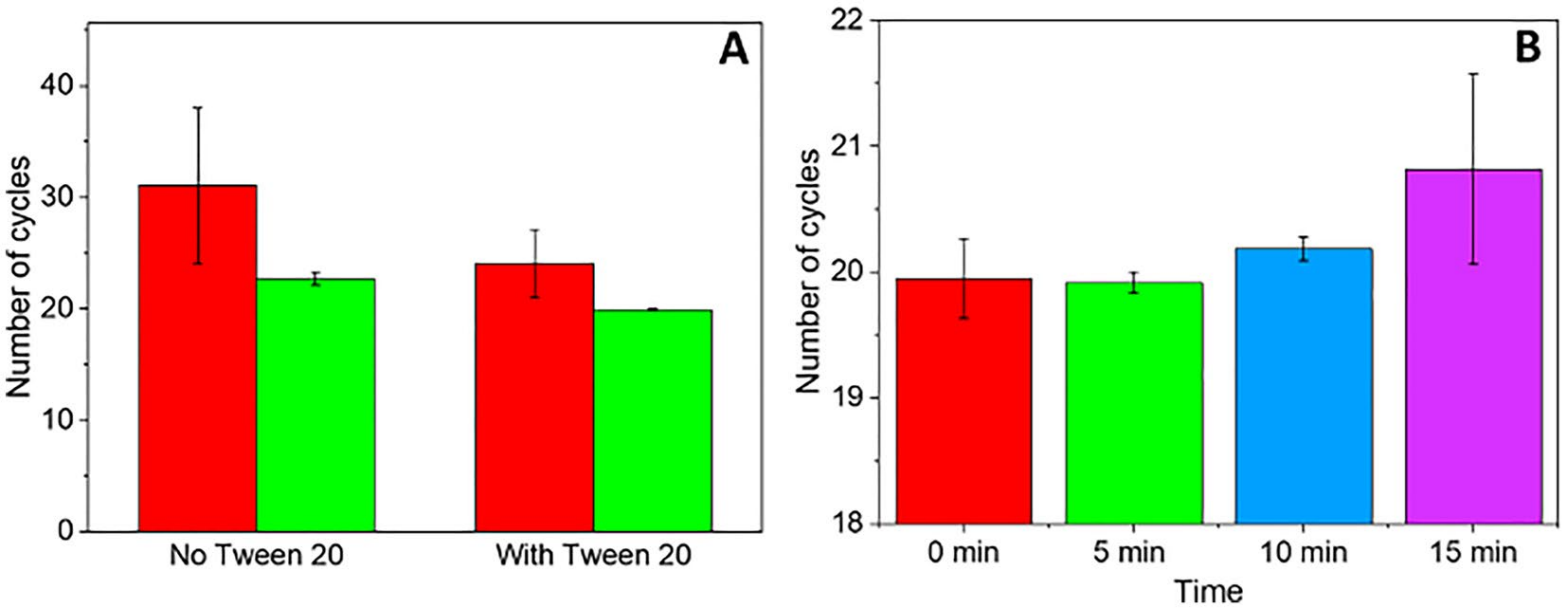
Optimisation of lysis conditions. (**A**) Cycle number following lysis with or without Tween 20 for *E. coli* (10^6^ CFU/mL). The inhibitory effect was compared without (red) and with washing step (green). (**B**) Cycle number for the eluate obtained for different lysis time in presence of Tween 20 and using a washing step (0–15 min).

As shown in Fig. 4A**,** the inclusion of Tween 20 led to a statistically significant enhancement in DNA concentration in the eluate, improving Cq values from 22.71 ± 0.59 to 19.91 ± 0.08 after washing or increasing the DNA concentration from 7.6 × 10^3^ to 61 × 10^3^ copies/µL (nearly 10 times). Previous studies also reported that Tween 20 is non-inhibitory in PCR reactions and can enhance the amplification efficiency under a range of concentrations (4—10%) by reducing inhibitory effects and stimulating *Taq* DNA polymerase activity^36–38^. This agrees with the modest drop in cycle number comparing with and without Tween 20, both with wash. Therefore, it is anticipated that the inhibition observed without washing in the presence of Tween 20 is the result of the salt, not the Tween 20. The duration of cell lysis was optimised by incubating a mixture of PASAP and cultured *E. coli* (10^4^ CFU/mL) at room temperature for different durations (0 to 15 min) before the DNA binding step using the MCE membrane. The measured DNA concentrations, as determined by qPCR, following elution are presented in Fig. 4B.

Overall, a trend of decreasing DNA concentration in the eluate was obtained with an increasing incubation period, with the amount of DNA detected drastically worsened when the *E. coli* mixture was incubated for 15 min. The denaturation of DNA can explain this by extended gDNA exposure time to the highly alkaline conditions (> pH 12)^39^. The extended lysis time could have damaged the DNA decreasing the amplification efficiency. Considering the 10-min wicking time and the similar DNA concentrations for 0- and 5-min incubation, it was concluded there was no need to for sample incubation with the PASAP reagent before starting the wicking, allowing combining lysis and binding steps into a single step.

### Effect of size of MCE paper on direct on-paper cLAMP

**Figure 5:**
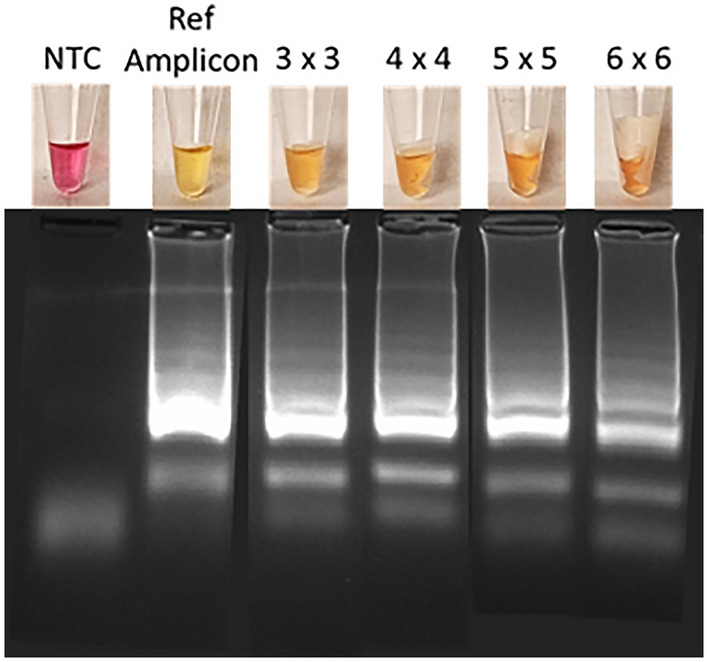
A cLAMP in presence of MCE paper. Squares with increasing size (3 mm × 3 mm, 4 mm × 4 mm, 5 mm × 5 mm, and 6 mm × 6 mm) were introduced into cLAMP of gDNA. Colorimetric change and cropped gel images for each paper size are compared with amplification without paper (Ref Amplicon) and the negative control (NTC). Image of the full gel is provided in the Supplementary Information.

Having demonstrated LAMP amplification in presence of 3 mm × 4 mm MCE paper by gel electrophoresis, it was confirmed the addition of phenol red allowed for cLAMP. The PASAP method was then used to bind 100 pg/µL DNA to the MCE paper, followed by wash with 70% ethanol, or no wash. The size of MCE paper that can be used for direct on-paper cLAMP is a compromise between binding capacity and attenuation of amplification. To optimise the paper size, MCE paper was cut into squares of 3 - 6 mm^2^. The squares were exposed to 20 µL PASAP lysis/binding reagent and with/without a wash with 70% ethanol for 30 s introduced to 20 µL LAMP reaction mix containing the gDNA. After heating to 68 °C for 50 min, amplification was visualised by the colour change and confirmed by gel electrophoresis, as shown in Fig. 5.

A colour change from pink to yellow/orange was observed for all paper sizes and gel electrophoresis confirmed that the colour change of LAMP reaction was indeed the result of amplification (Fig. 5). However, from the intensity of gel bands, it is apparent that the quantity of amplified DNA decreased gradually when increase the size of the MCE paper. This agrees with an earlier report using 2-mm glass fibre discs placed in 50 µL cLAMP mix for the detection of down to 10^3^ copies /mL of rotavirus A within 30 min, when larger discs would inhibit amplification^40^. Here, cutting the brittle MCE paper with scissors limited the size to strips that were 3—4 mm wide. Recognising this is a crude and manual approach, scissors were selected after we found a laser cutter burned the edges, impeding wicking efficiency and a office paper guillotine fragmented the membrane in irregular sizes. The importance of the optimised washing for cLAMP was confirmed conducting experiments with 100 pg/µL gDNA with and without the 10 s wash in 40% IPA. The resulting colour changes and gels are shown in the supplementary information (Fig. SI 5), showing significant attenuation without washing as a result of inhibition. Note that while a positive was detected after 30 min for a high concentration gDNA sample, decreasing the amplification time would lead to a decrease in sensitivity.

The benign PAPAS reagent and limited supplies (a pipette with 2 pipette tips, 2 test tubes and the MCE strip) make the PASAP method attractive for in-field testing, particularly in low resource settings. With all amplifications listed above conducted in a PCR machine, compatibility with in-field use was confirmed using a purpose-designed heater, accommodating the PCR vials in recesses CNC-milled in an aluminium block (Fig. S9A,B). The temperature was controlled using a laptop using the feedback from a temperature sensor. Evaporation of the amplification mixture when heated in the aluminium block prevented amplification, probably because unlike in PCR machines the block did not have a heated lid, leading to condensation. A drop (~ 20 μL) of silicone oil provided an effective inert barrier to prevent evaporation of the amplification solution. Preliminary testing of the 12 V heater yielded following PASAP of a gDNA standard yielded comparable results to those obtained using the PCR instrument, showing a colour change from yellow to pink following 60 min at 68 °C for 100 pg/µL gDNA (Fig. S9C). No differences were found between the heater powered using a power supply or battery pack, conforming suitability for in-field use.

### Assay sensitivity and *E. coli* detection in milk

With the PASAP/cLAMP method established, *E. coli* was used as biological target to understand applicability, and milk was selected as sample matrix. Recently, multienzyme isothermal rapid amplification was used for the detection of *Streptococcus* in milk following its extraction on filter paper following lysis^41^. Here, cultured bacteria were serially diluted in PBS from 10^4^ to 10^1^ CFU/mL, and 50 µL of the diluted samples were used for the PASAP method following the optimised workflow with negative control (fresh PBS without *E. coli*).

**Figure 6:**
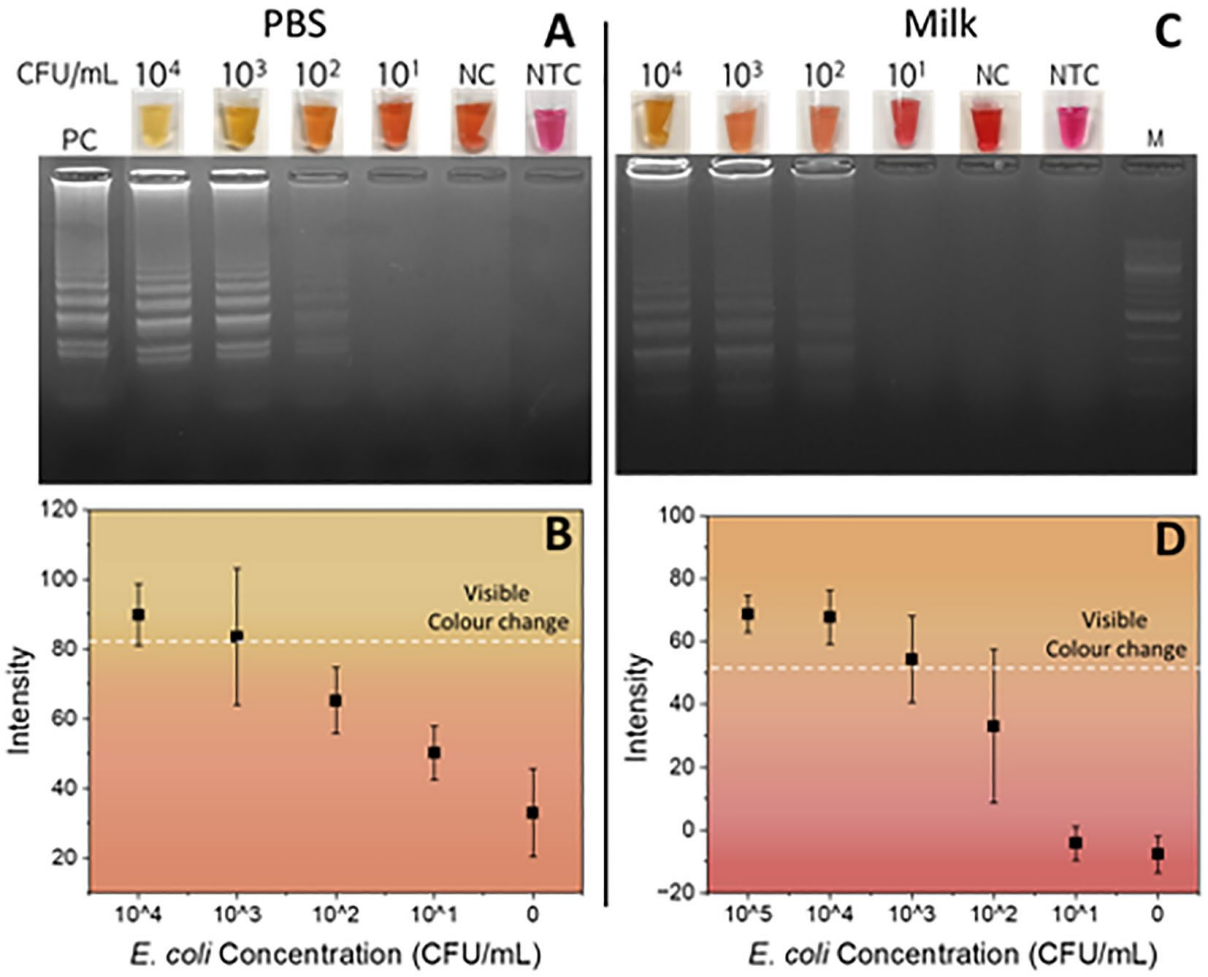
Assay sensitivity for the detection of *E. coli* (n = 3) at concentrations ranging from 10 to 10^4^ CFU/mL was indicated, NC and NTC represent negative control and no template control, respectively. (**A**) Colorimetric change and cropped gel images for *E. coli* detection in PBS and (**B**) intensity plot for *E. coli* detection in PBS*.* (**C**) Colorimetric change and cropped gel images *E. coli* detection in skim milk and (**D**) intensity plot for *E. coli* in milk. The intensity measurement was inspired by earlier study^29^. The white dashed line in (**B**) and (**D**) indicates the visible colour changes of the reaction tubes by the naked eye. Full gel images are supplied in Supplementary Information.

The PASAP method combined with cLAMP successfully allowed for the detection of *E. coli* from PBS solutions with a LOD at 10^2^ CFU/mL, or 1 CFU present in the 10 µL sample, with the presence of amplified DNA for all positive colourimetric results confirmed by gel electrophoresis (Fig. 6A). Semi-quantitative data were obtained by image analysis^29^, (Fig. 6B), showing the calculated colour intensity of LAMP solution for a range of *E. coli* concentrations**.** We previously reported that 15 CFU/50 µL could be detected using a magnetic bead kit, and 150 CFU/50 µL using a spin column^23^, making 1 CFU/10 µL slightly better than but inferior to the 0.15 CFU detected in a 50 µL using the ASAP method. This may due to decreased binding strength as a result of the lower zeta potential of the MCE paper (− 7 to − 10 mV^42^ vs − 14 mV^43^), or less favourable binding kinetics in the flow induced by the wicking.

When using milk as the sample, the yellow hue indicative of a positive test shifted slightly towards orange, but a change from the pink in the absence of DNA could still observed at 10^2^ CFU/mL; an evident change was only found at 10^3^ CFU/mL (Fig. 6C). While detection through colour change and gel bands at 10^2^ CFU/mL was demonstrated twice in two of five replicates, the high failure rate means, using the PASAP method, E. *coli* can be detected in milk at 10^3^ CFU/mL (Fig. 6D). This decreased sensitivity compared with other work^14,41^ should be contrasted by a significant reduction in reagents, consumables, waste and manual handling. The sensitivity of PASAP/cLAMP may be improved by increasing the sample volume processed, and future research will focus on the introduction of a wicking pad to increase the sample volume, as currently the sample volume is limited by low wicking rate of the PASAP solution in the MCE paper. Membranes combining high wicking rate with a higher charge density can also be investigated. DNA precipitation under crowding conditions at high salt have been demonstrated for moderate charge density of carboxylic acid functionalised surfaces. It should be noted that the surface plays an important role, as DNA binding to silica surfaces relies on a different binding mechanism (electrostatic and hydrophobic interaction) and does not require a crowding agent, and is typically more effective at decreased charge density at neutral pH^44^.

The presented PASAP method provides a fast, low-cost and easy sample preparation method compatible with on-paper cLAMP that can be applied to complex biological samples, including for the detection of bacteria in milk. The simple workflow is suitable for automation and  when combined with a battery-powered heater has potential to be integrated into hand-held-devices for PONT in food safety and diagnostics.

## Conclusions

A new method for on-paper DNA extraction and cLAMP with minimal use of reagents, consumables and equipment is presented. The aqueous alkaline PEG-based reagent lyses the cell, providing the high pH for alkaline lysis, and enhancing the lytic activity with the surfactant Tween 20. Owing to crowding conditions in presence of PEG and NaCl, the DNA was immobilised at the tip of a strip of mixed cellulose ester paper used for wicking the lysate. Following a 10-s wash conducted by dipping the strip in 40% isopropanol, the tip of the dried paper strip is snapped off into a vial for colourimetric LAMP. The proposed workflow takes 15 min and uses wicking for liquid handling. The instrumentation used was minimal (a pipette for depositing 20 μL drops of sample and washing reagent) and produced minimal waste (2 vials, 2 pipette tips and a 3 mm × 45 mm membrane). Supporting the isothermal amplification with a battery-powered 12 V heater (60 min at 68 °C), the method is compatible with in-field use. Recognising the quantitative limitations of cLAMP, the method allowed for the detection of *E. coli* down to 10 CFU/mL in buffer, increasing to 10^3^ CFU/mL in 1:10 diluted milk; though, occasionally a colour change was detected at 10^2^ CFU/mL. Future work will focus on enhancing the sensitivity by processing larger sample volumes without compromising processing time, for example, connecting the paper with an absorbent pad.

### Supplementary Information

Supplementary Figures.

## Data Availability

The data generated and/or analysed during the current study not presented in this paper are available from the corresponding author on reasonable request.
