# Supplementary material for: Method for lysis and paper-based elution-free DNA extraction with colourimetric isothermal amplification
Source: Sci Rep. 2024 Jun 24;14:14479. doi: 10.1038/s41598-024-59763-4 (PMC11196276; doi:10.1038/s41598-024-59763-4)
Supplement: Supplementary file 1 — Supplementary Figures. [file 41598_2024_59763_MOESM1_ESM.docx]

Method for Lysis and Paper-based Elution-free DNA Extraction with Colorimetric Isothermal Amplification

**Soo Min Lee ^1^, Egan H. Doeven ^1,2^, Dan Yuan ^1,3*^, Rosanne M. Guijt ^1*^**

^1^ Deakin University, Centre for Regional and Rural Futures (CeRRF), Locked Bag 20000, Geelong, VIC 3220, Australia

^2^ Deakin University, School of Life and Environmental Sciences, Faculty of Science, Engineering and Built Environment, Waurn Ponds, VIC 3216, Australia

^3^ The University of Queensland, School of Mechanical and Mining Engineering, Brisbane, QLD 4072, Australia

*Corresponding author email: [rosanne.guijt@deakin.edu.au](mailto:rosanne.guijt@deakin.edu.au)

SI-1 Selection of MCE membrane


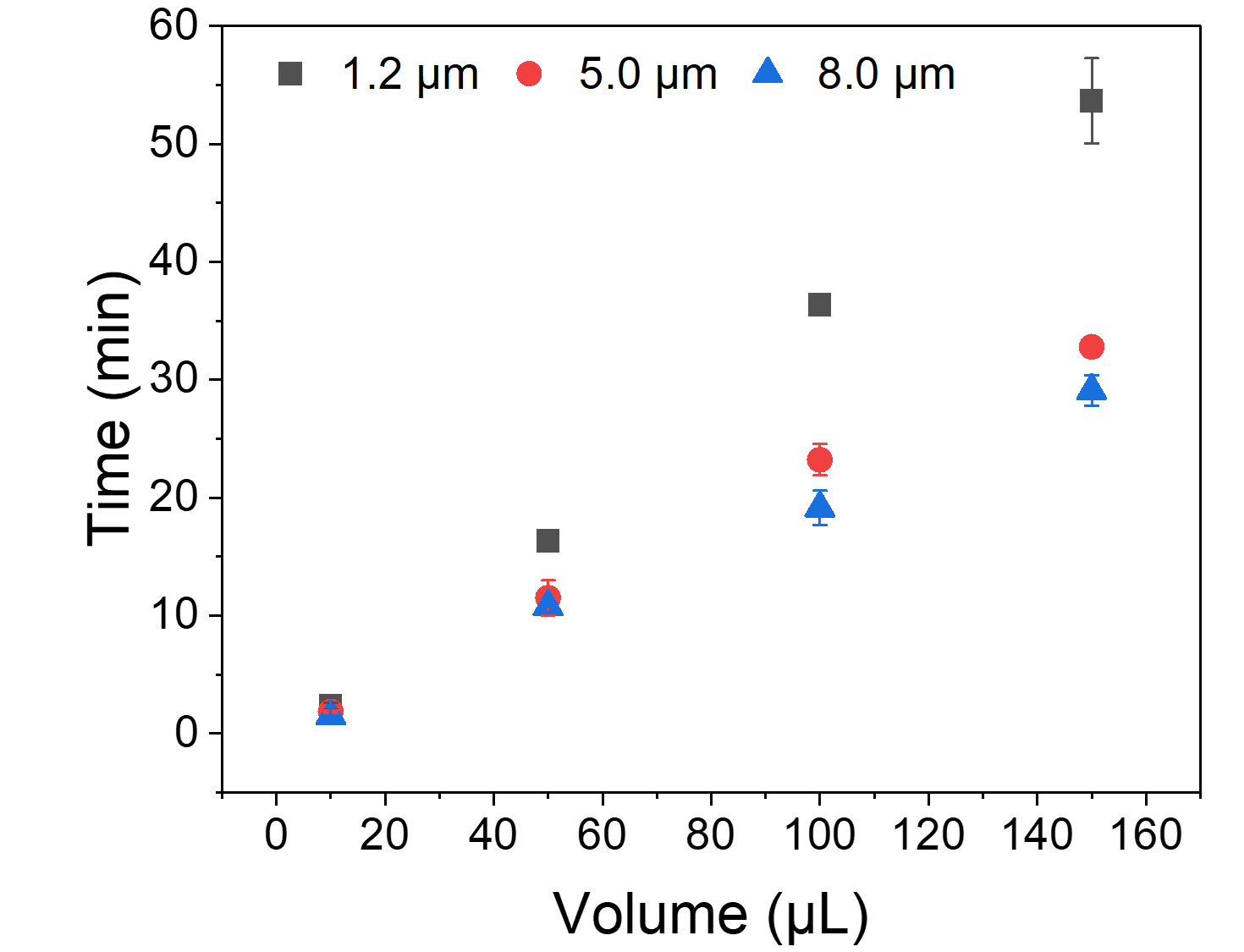


Figure S 1**.** Time for wicking 10, 50, 100, and 150 µL PASAP solution using MCE papers with different pore sizes: 1.2 µm (black), 5.0 µm (red), and 8 µm (blue).

SI-2 Amplification in presence of MCE membrane

qPCR and qLAMP were tested to determine the compatibility with MCE paper. The PASAP method was performed with purified gDNA as target analyte. The amplification curve shown in Figure S 2 indicates no amplification product was formed detected regardless of washing when the MCE paper was presented during amplification. In contrast, when the PASAP method was followed by wash and with elution, target DNA was amplified. Further analysis by gel electrophoresis and the melting curve agreed no PCR amplification in presence on the MCE paper, and only eluted DNA was amplified.


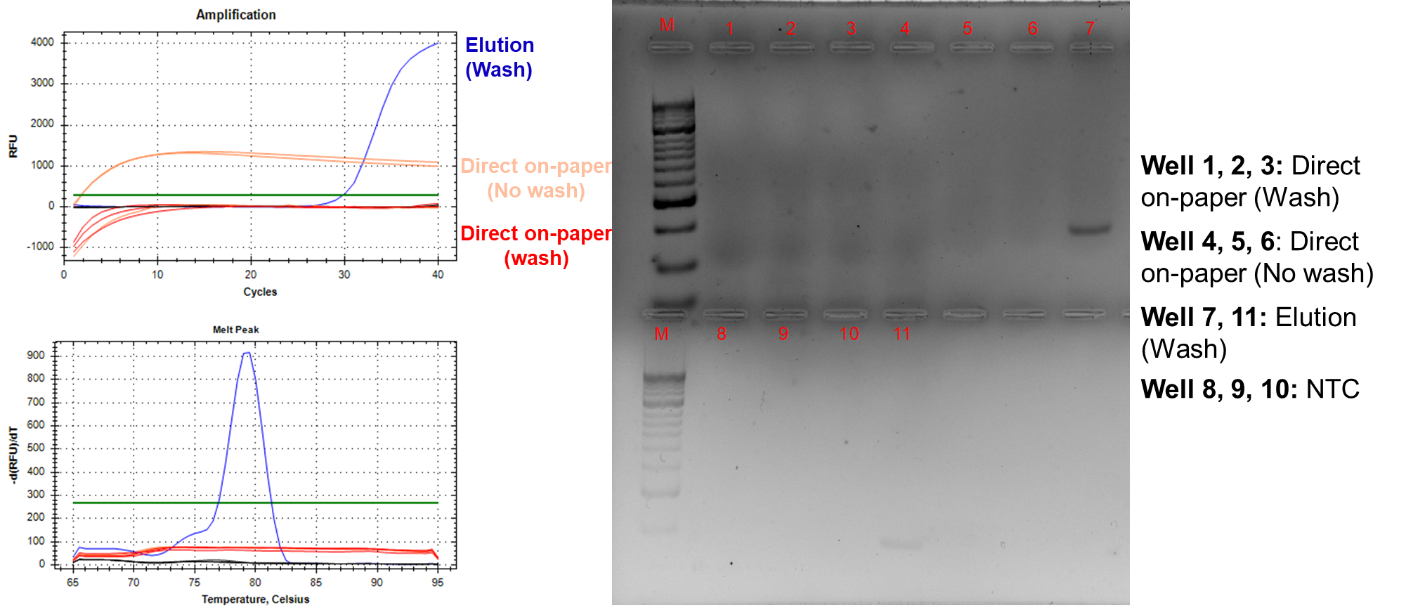


Figure S 2 qPCR amplification curves, melting curve, and gel electrophoresis Well 1, 2, 3, : direct on-paper (after washing), well 4, 5, 6: direct on-paper (before washing), well 7, 11: elution (after washing), and well 8, 9, 10: NTC.

Similarly, during qLAMP gDNA was only detected in the eluate. However, amplicons were found in the amplification mixture when paper was present during amplification (with and without wash), as evidenced by the bands on the agarose gel as shown in Figure S 3


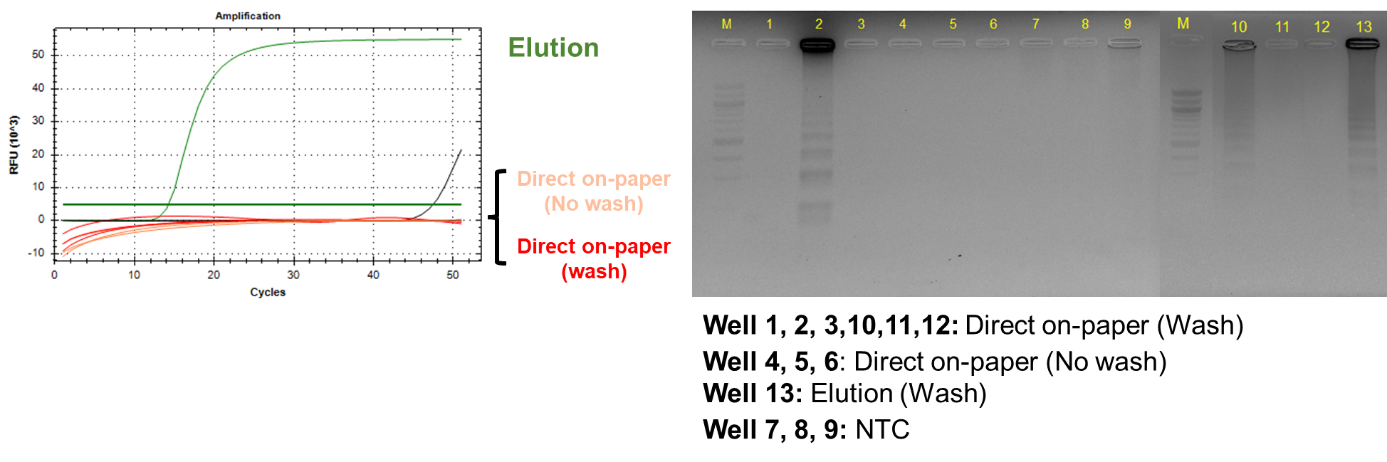


Figure S 3 Amplification curve of qLAMP and gel electrophoresis of amplicons. Well 1, 2, 3, 10 ,11, 12: direct on-paper (after wash), well 4, 5, 6: direct on-paper (before wash), well 13: elution (after wash), and well 7, 8, 9: NTC

To optimise the washing solvent, the IPA content was increased from 0 to 100%. For IPA <40%, a trend towards higher Cq values with longer washing times suggests the solubility of the DNA is too high and DNA is lost during the wash. Reversibly, more efficient washing for IPA>40% is found for longer washing times, suggesting the poorly soluble DNA remains bound to the paper while inhibitors are removed. Other than the results for 100 % IPA, the differences observed were not statistically significant, but the lowest Cq value in the shortest time was obtained after a 10 sec wash in 40% IPA.

## qPCR Standard Curve


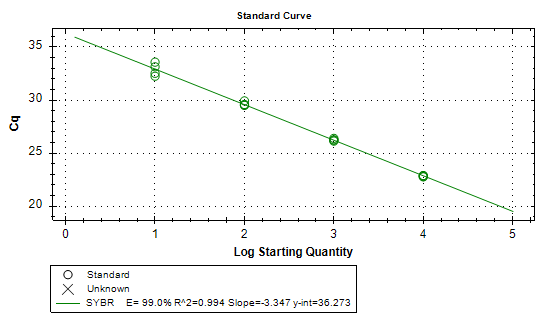


Figure S 4 A standard curve generated using purified gDNA of *E.coli* BL21 using commercial spin-column DNA extraction kit (ISOLATE II genomic DNA extraction kit). All samples where tested in triplicates. The amplification efficiency (E) was 99.0 % with R^2^ of 0.994. Using each value of slope (-3.347) and y-intercept (36.273), an equation was constructed as Y = -3.347 X + 36.273 where Y = Cq value, and X = log (DNA copy number/reaction). From ^1^

Figure S 5 Cycle number obtained in the eluate for washing with increasing isopropanol content


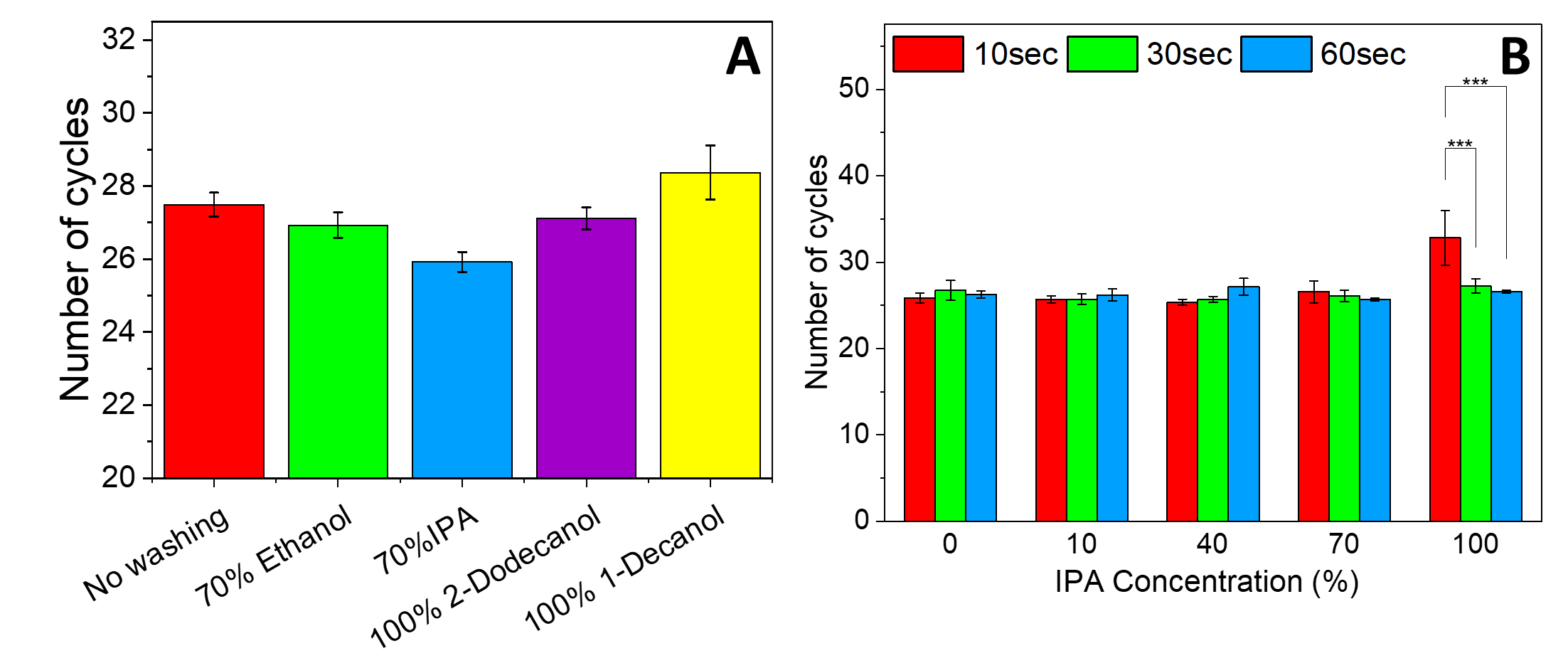


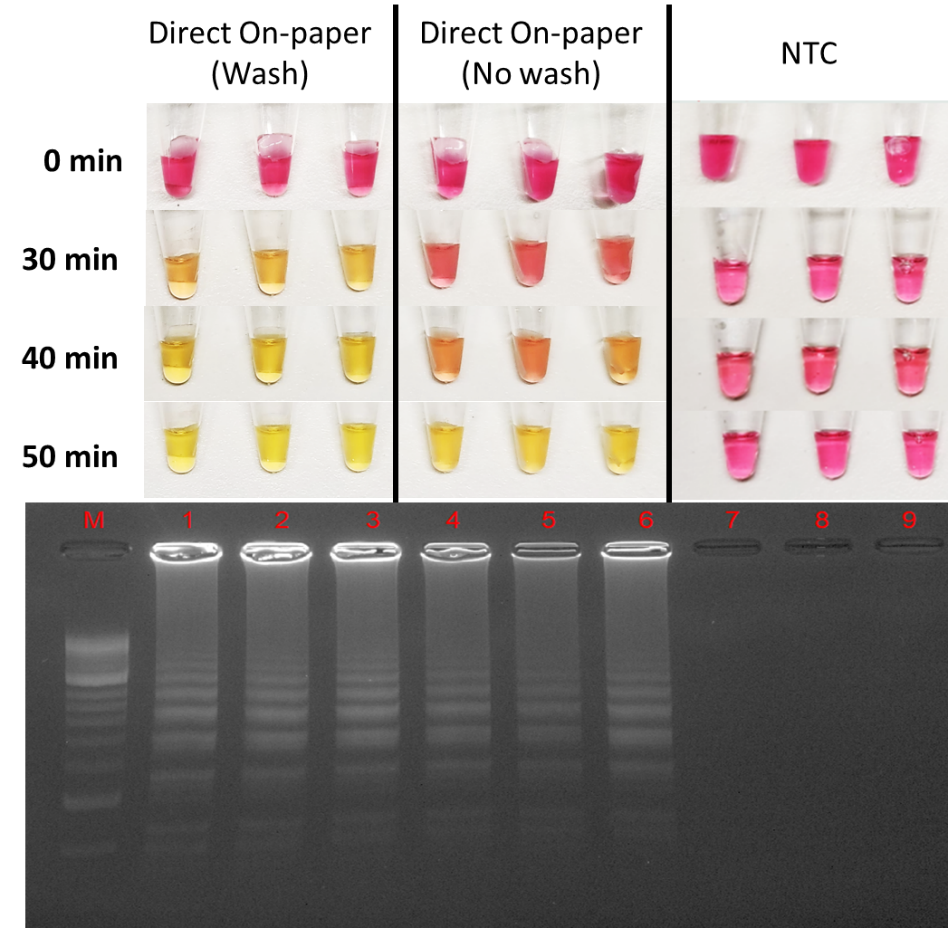


Figure S 6 Photographs for phenol red-based colourimetric LAMP integrated with the PASAP method (top) and gel electrophoresis (bottom) of amplification products 100 pg/µL gDNA (n=3). Lane 1,2,3: PASAP with washing; Lane 4, 5, 6: PASAP without washing; and Lane 7, 8, 9: No template control (NTC).


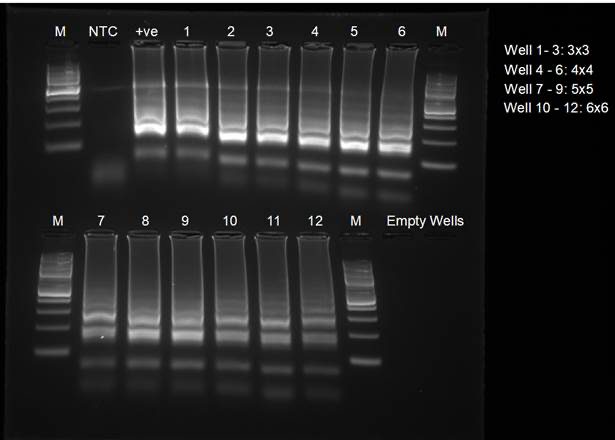


Figure S 7 Full gel image for Figure 6


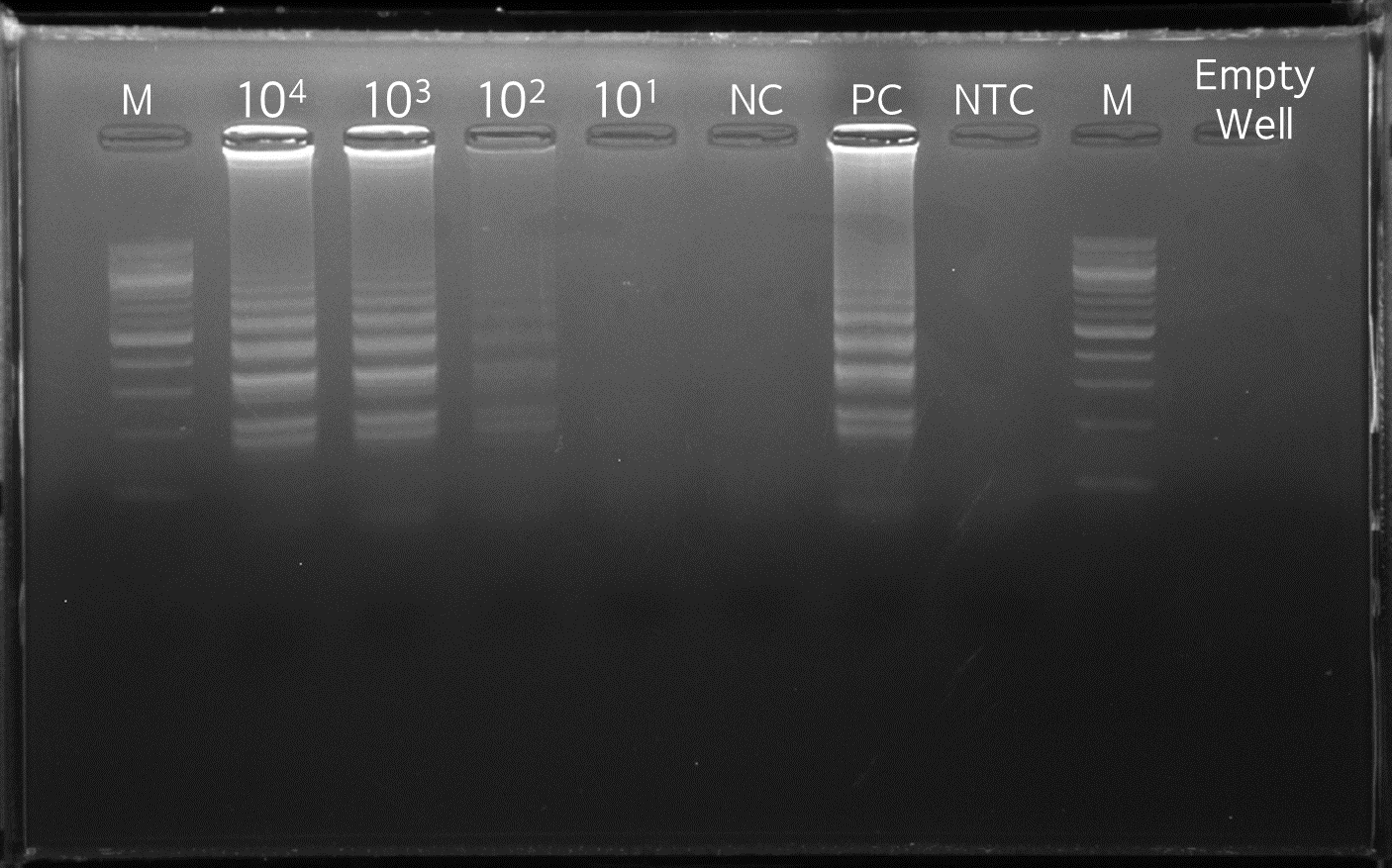


Figure S 8 Full gel for Figure 7b


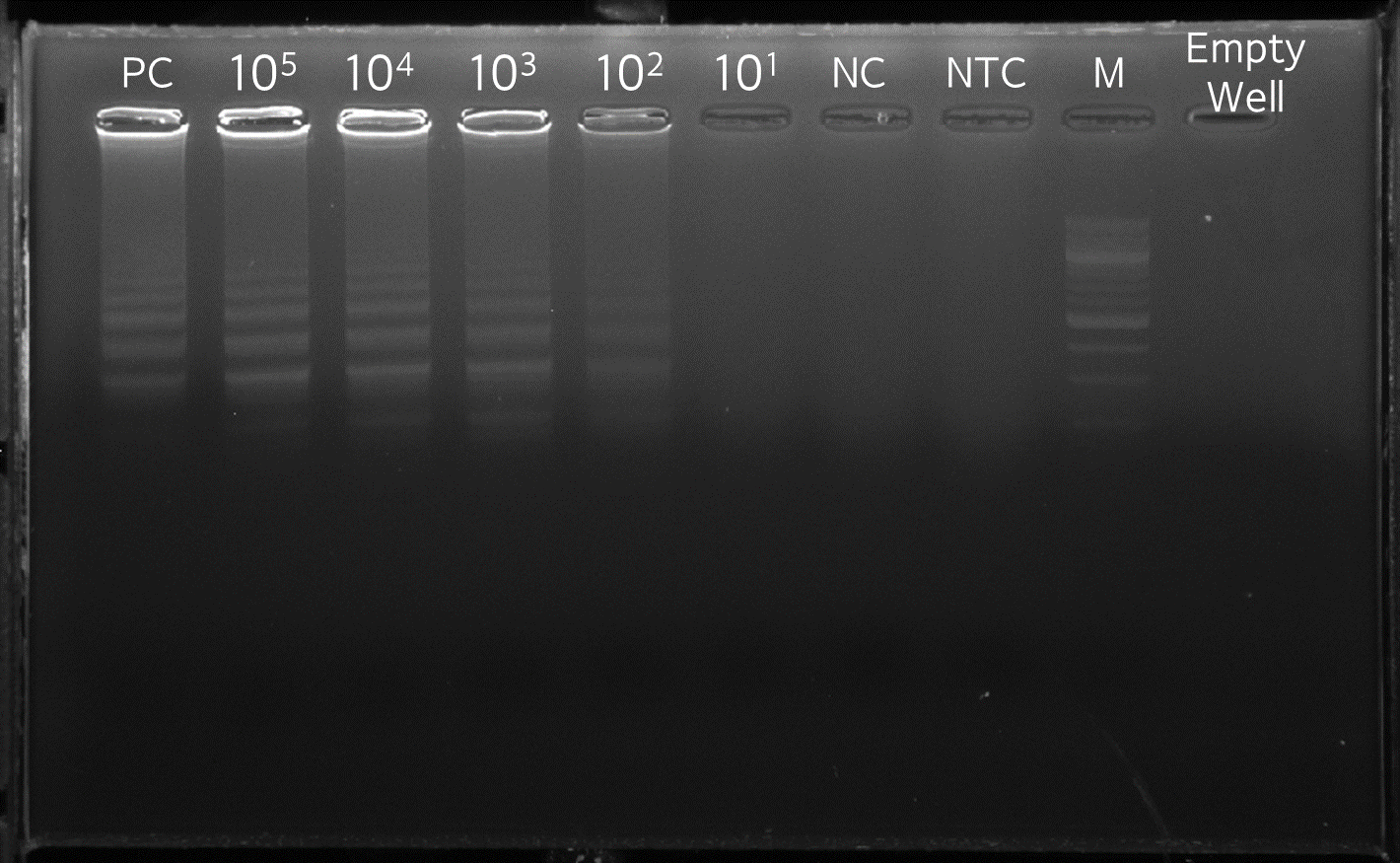


Figure S 9 Full gel for Figure 7c

SI 3 Battery operated heater

To confirm suitability of the PASAP/cLAMP assay for use outside a laboratory, the method was tested using a custom 12V heater, as shown in Figure S 6


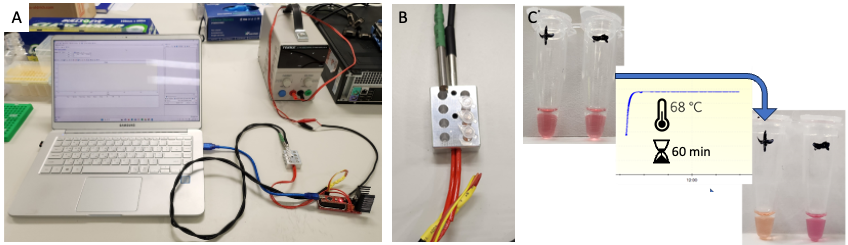


Figure S 10 PASAP/cLAMP using 12 V heater, powered using a power supply. A. The set-up. B The aluminium block connected to the heater and sensor that could be used for up to 8 vials. C. cLAMP following PASAP of gDNA (+) and negative control (-).

1. Lee, S.M., Balakrishnan, H.K., Yuan, D., Nai, Y.H., and Guijt, R.M. (2021). Perspective - what constitutes a quality analytical paper: Microfluidics and Flow analysis. Talanta. Open. *4*, 100055. 10.1016/j.talo.2021.100055.
